# “Any Condomless Anal Intercourse” is No Longer an Accurate Measure of HIV Sexual risk Behavior in Gay and Other Men Who have Sex with Men

**DOI:** 10.3389/fimmu.2015.00086

**Published:** 2015-02-27

**Authors:** Fengyi Jin, Garrett P. Prestage, Limin Mao, I. Mary Poynten, David J. Templeton, Andrew E. Grulich, Iryna Zablotska

**Affiliations:** ^1^The Kirby Institute, University of New South Wales, Kensington, NSW, Australia; ^2^The Centre for Social Research in Health, University of New South Wales, Kensington, NSW, Australia; ^3^RPA Sexual Health, Sydney Local Health District, Sydney, NSW, Australia

**Keywords:** condomless anal intercourse, HIV risk, homosexuality, male, cohort study, Australia

## Abstract

**Background:** Condomless anal intercourse (CLAI) has long been recognized as the primary mode of sexual transmission of HIV in gay and other men who have sex with men (MSM). A variety of measures of CLAI have been commonly used in behavioral surveillance for HIV risk and to forecast trends in HIV infection. However, gay and other MSM’s sexual practices changed as the understanding of disease and treatment options advance. In the present paper, we argue that summary measures such as “any CLAI” do not accurately measure HIV sexual risk behavior.

**Methods:** Participants were 1,427 HIV-negative men from the Health in Men cohort study run from 2001 to 2007 in Sydney, Australia, with six-monthly interviews. At each interview, detailed quantitative data on the number of episodes of insertive and receptive CLAI in the last 6 months were collected, separated by partner type (regular vs. casual) and partners’ HIV status (negative, positive, and HIV status unknown).

**Results:** A total of 228,064 episodes of CLAI were reported during the study period with a mean of 44 episodes per year per participant (median: 14). The great majority of CLAI episodes were with a regular partner (92.6%), most of them with HIV-negative regular partners (84.8%). Participants were more likely to engage in insertive CLAI with casual than with regular partners (66.7 vs. 55.3% of all acts of CLAI with each partner type, *p* < 0.001). Men were more likely to report CLAI in the receptive position with HIV-negative and HIV status unknown partners than with HIV-positive partners (*p* < 0.001 for both regular and casual partners).

**Conclusion:** Gay and other MSM engaging in CLAI demonstrate clear patterns of HIV risk reduction behavior. As HIV prevention enters the era of antiretroviral-based biomedical approach, using all forms of CLAI indiscriminately as a measure of HIV behavioral risk is not helpful in understanding the current drivers of HIV transmission in the community.

## Introduction

Soon after the emergence of HIV/AIDS more than three decades ago (1), having anal intercourse without a condom, known as unprotected anal intercourse (UAI), was recognized as the key HIV transmission route in gay and other men who have sex with men (2–4). A strong association between UAI and HIV infection in gay and other men who have sex with men has been reported at both individual and population levels (5). Recently, the introduction of a broader range of biomedical prevention strategies prompted the US Centers for Disease Control and Prevention to recommend a change in terminology from UAI to condomless anal intercourse (CLAI) (6), to reflect the fact that much condomless intercourse is not “unprotected” form the point of view of HIV transmission (7).

In the early stage of the HIV/AIDS epidemic when a diagnosis of HIV infection represented a death sentence, gay male communities in developed countries embraced a concept of “safer sex,” which entailed the use of condoms for anal intercourse and the avoidance of any type of CLAI. In this pre-treatment era, dramatic reductions in CLAI (8–11) led to major declines in HIV transmission in this population group (12–14). Measures of CLAI became widely used as a marker of high-risk sexual behavior and were targeted in health promotion and education campaigns to reduce sexual transmission of HIV among gay and other men who have sex with men. A variety of measures of CLAI have been commonly used in behavioral surveillance for HIV risk and to forecast trends in HIV infection (5, 15).

In the subsequent decades, sexual practices changed in parallel to development of successful HIV therapy and improved knowledge of HIV transmission. In the mid-1980s, the introduction of the HIV serological test first enabled the diagnosis of HIV-infected individuals. Later, detailed behavioral research of per-contact risk of CLAI provided evidence of the relatively lower likelihood of acquiring HIV when the HIV-negative partner took the insertive role during CLAI (16, 17). The advent of highly active antiretroviral therapy (ART) in the mid-1990s and its continuing improvement transformed the perception and the reality of a diagnosis of HIV infection from a death sentence to a manageable chronic condition (18). As a result, the reductions in CLAI observed in 1980s were not maintained at the same level over time, but rather have been replaced by a range of so called “risk reduction practices,” particularly in gay male communities with high levels of knowledge about HIV (19, 20). These behaviors involve an individual choosing to engage in anal intercourse without a condom in situations where he believes the risk of HIV transmission is reduced if not completely eliminated by use of risk reduction approaches other than condoms.

At least six forms of HIV risk reduction practices have been described. By the mid-2000s, four HIV risk reduction behaviors have been described focusing on the knowledge on partner’s HIV status. Serosorting is when CLAI is practiced only with partners believed to be of the same HIV status (21–23). Negotiated safety is a form of serosorting where members of HIV-negative couples in a regular relationship each test for HIV early in their relationship, agree not to have CLAI outside their relationship, and then agree to have CLAI with each other (24, 25). Strategic positioning is when a HIV-negative man takes only the insertive role during CLAI, and an HIV-positive man takes the receptive role (26). During CLAI, withdrawal is when the insertive partner withdraws his penis out of the HIV-negative partner’s rectum before ejaculation occurs. Later on, two more practices have emerged utilizing information on HIV viral load and HIV treatment. Viral load sorting is when a HIV-negative man engages in CLAI with a HIV-positive partner only when the positive partner’s HIV viral load is undetectable (27). HIV pre-exposure prophylaxis, known as PrEP, is the HIV-negative man taking ART on a regular basis to minimize the risk of HIV transmission when engaging in CLAI (7).

The adoption of these risk reduction behaviors by some gay men has been accompanied by an increase in overall reports of CLAI since the mid-1990s (11, 28). Some countries have adapted to use more specific forms of CLAI, which represent a higher risk for HIV transmission to monitor trends in sexual behavioral risk. In countries like Australia and US, CLAI with casual partners is differentiated from CLAI with regular partner (29, 30). In UK, levels of CLAI with non-seroconcordant partners were measured as higher behavioral risk (31).

Historically, CLAI has been used as a primary indicator of risk behavior for both surveillance and research purposes. However, in the most recent stages of the HIV epidemic, many measures of CLAI fail to reflect the complexity of sexual behavior and to discriminate sexual risk behavior from risk reduction practices among gay and other men who have sex with men. In this paper, we use data from a cohort of HIV-negative gay and other men who have sex with men to demonstrate that CLAI is often practiced in an evidence-informed and considered manner by many gay and other men who have sex with men. We use detailed analyses of reported CLAI episodes to argue that summary measures such as “any CLAI” are no longer an accurate measure of HIV sexual risk behavior. As the study commenced in 2001, we focus our analysis on behaviors mostly relevant to serosorting, strategic positioning, and withdrawal.

## Materials and Methods

The Health in Men study was a prospective cohort study of HIV-negative gay and other men who have sex with men recruited from community-based settings in Sydney, Australia (32). Men were eligible if they met the following criteria: (1) reported having sex with other men within the previous 5 years, (2) lived in Sydney or participated regularly in its gay community, and (3) tested HIV-negative at baseline. The study was approved by the Human Research Ethics Committee at the University of New South Wales.

From June 2001 to December 2004, the study recruited a total of 1,427 initially HIV-negative men and they underwent follow-up interviews every 6-months after baseline interview till June 2007. At each interview, detailed quantitative data on the number of episodes of insertive and receptive CLAI in the last 6 months were collected for regular and for casual partners, by perceived HIV status of these partners (negative, positive, or unknown), and, for receptive CLAI, by whether or not ejaculation inside participants’ rectum occurred. Episodes of anal intercourse involving condom failures (e.g., condom breakage and slippage) were included as episodes of CLAI of each relevant mode, and were not separately recorded. This detailed analysis included all episodes of CLAI reported to take place between the first follow-up interview and the end of study for those who remained HIV-negative, and to the estimated date of HIV seroconversion for those who became HIV-infected during the study (33). All episodes of CLAI reported at baseline were excluded as these CLAI events occurred prior to the commencement of the study. Participants also reported their primary regular partner’s HIV viral load at each study interview.

### Risk reduction behaviors

Risk reduction behaviors including serosorting, strategic positioning, and withdrawal were derived from participants’ reports about modes of CLAI, and were defined in detail elsewhere (34). However, participants’ conscious intent to practice these risk reduction behaviors was not assessed. Briefly, serosorting was defined as reporting CLAI at study interviews, and all CLAI were with partners who were reported by study participants to be HIV-negative; strategic positioning was defined as reporting CLAI at study interviews, and all CLAI were insertive, and withdrawal was defined as reporting receptive CLAI at study interviews, and that none of the receptive CLAI had involved ejaculation inside the rectum.

### Statistical analysis

Statistical analyses were performed using STATA 13.1 (STATA Corporation, College Station, TX, USA). Trends in behavioral change during the study period, including any CLAI, serosorting, strategic positioning, and withdrawal were analyzed using logistic regression. Chi square tests were used to compare the proportions of different modes of CLAI in relation to partner’s HIV status.

## Results

The median age of participants at enrollment was 35 years, ranging from 18 to 75 years. The vast majority (95.2%) of participants self-identified as gay or homosexual. More than half (57.8%) reported a current regular partner at baseline. The overall follow-up time was 5,160 person-years contributed by 1,381 (96.8%) men who completed at least one follow-up interview, with a median of 3.9 years per participant.

During the study period, a total of 228,064 episodes of CLAI were reported (Table 1), giving a mean of 44 episodes (SD: 73) of CLAI per year per participant, with a median number of 14 episodes (interquartile range: 1–59). Each year, the proportion of men who reported any CLAI varied from 63.3% in 2001 to 57.3% in 2007 (*p* trend = 0.132). Overtime, the proportion of participants who practiced serosorting with any sexual partners increased significantly from 48.2% in 2001 to 71.5% in 2007 (*p* trend < 0.001). The proportion of participants who practiced strategic positioning remained stable at around 25% each year (*p* trend = 0.930), so did the proportion of participants who practiced withdrawal at round 20% (*p* trend = 0.998).

**Table 1:** **Number of episodes of condomless anal intercourse by participants’ reported type and HIV status of partner in the Health in Men study**.

CLAI by sexual positioning	HIV-negative partners			HIV-positive partners			HIV status unknown partners			Total	
	Number of men	Number of episodes	%	Number of men	Number of episodes	%	Number of men	Number of episodes	%	Number of episodes	%
With regular partners
Insertive CLAI	818	105,485	46.3	99	6,869	3.0	179	4,326	1.9	116,680	51.2
Receptive CLAI with withdrawal	711	32,273	14.2	58	1,749	0.8	126	1,412	0.6	35,434	15.5
Receptive CLAI with ejaculation	635	55,696	24.4	25	583	0.3	89	2,693	1.2	58,972	25.9
Subtotal		193,454	84.8		9,201	4.0		8,431	3.7	211,086	92.6
With casual partners
Insertive CLAI	293	2,897	1.3	80	1,174	0.5	487	7,257	3.2	11,328	5.0
Receptive CLAI with withdrawal	203	1,249	0.5	40	261	0.1	325	2,441	1.1	3,951	1.7
Receptive CLAI with ejaculation	113	818	0.4	10	25	0.01	133	856	0.4	1,699	0.7
Subtotal		4,964	2.2		1,460	0.6		10,554	4.6	16,978	7.4
Total										228,064	100

*CLAI, condomless anal intercourse*.

*Number of men who reported at least one episode of such act. Percentages are of the total number of episodes of CLAI (228,064)*.

**Figure 1:**
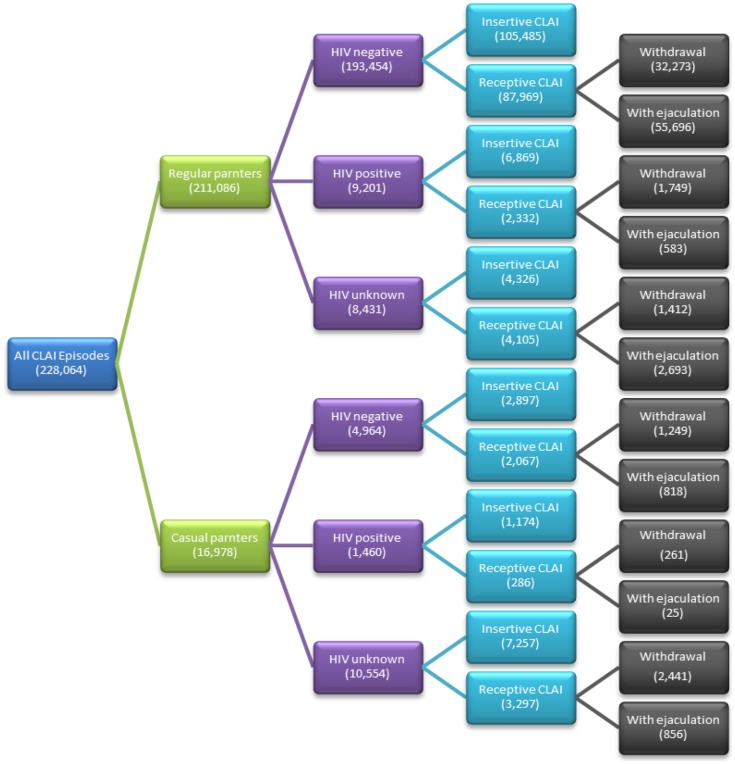
**Proportions of condomless anal intercourse by partners’ HIV status, partner type, and sexual positioning in the Health in Men study**. CLAI, condomless anal intercourse.

The great majority of CLAI episodes (92.6%) were with a regular partner. CLAI with a HIV-negative regular partner accounted for 84.8% of the total CLAI episodes, and around 4% each were reported with HIV-positive and HIV status unknown regular partners (Figure 1). With a HIV-positive regular partner, only 25.4% of the total CLAI episodes were in the receptive position, and this was significantly lower than the proportion of receptive CLAI when the regular partner was HIV-negative (45.5%, *p* < 0.001) or HIV status unknown (48.7%, *p* < 0.001). Further, during receptive CLAI with a HIV-positive regular partner, only 25.0% of episodes involved ejaculation inside the participants’ rectum, which was also significantly lower than receptive CLAI with HIV-negative (63.3%, *p* < 0.001) and HIV status unknown partners (65.6%, *p* < 0.001).

Only 7.4% of the total CLAI episodes were with casual partners, and the majority of CLAI episodes were with casual partners of unknown HIV status (Table 1). About two-thirds (66.7%) of the total CLAI episodes with casual partners were in the insertive position, and this was significantly higher than the proportion of CLAI episodes that were in the insertive position with regular partners (55.3%, *p* < 0.001). Regardless of partners’ HIV status, most receptive CLAI with casual partners involved withdrawal before ejaculation (69.9%). When engaging in receptive CLAI with casual partners, only 8.7% of episodes involved ejaculation when the partner was HIV-positive, which was significantly less than that with HIV-negative casual partners (39.6%, *p* < 0.001) and with HIV status unknown casual partners (26.0%, *p* < 0.001).

At baseline, 79 participants (5.5%) reported a HIV-positive primary regular partner. Among them, 31 (39.2%) reported the HIV-positive partner having undetectable viral load, 21 (26.6%) whose partner had detectable viral load, and the rest 27 (34.1%) did not have knowledge of their partner’s viral load. During follow-up, a total of 7,618 episodes CLAI were reported with HIV-positive primary regular partners. Among them, 4,492 (59.0%) episodes occurred when the partner had undetectable viral load, 671 (8.8%) episodes were reported when the partner had detectable viral load, 2,455 (32.2%) episodes occurred when the partner’s viral load was unknown to the participants.

## Discussion

This detailed examination of nearly a quarter of a million of CLAI episodes reported by HIV-negative gay and other men who have sex with men in Sydney clearly indicates patterns of HIV risk reduction behavior. These patterns are (1) a high proportion (92.6%) of total CLAI episodes being with regular rather than casual partners; (2) for CLAI with regular partners, a high proportion of CLAI with partners of the same HIV status, indicative of serosorting; (3) a high proportion of insertive CLAI, indicative of strategic positioning, even more so when engaging in CLAI with casual partners; and (4) a high proportion of receptive CLAI involving withdrawal before ejaculation with non-HIV-negative partners. In fact, only 1.8% (*n* = 4,157) out of the total of 228,064 episodes of CLAI reported over a period of 7 years involved receptive CLAI with ejaculation inside the rectum with a non-HIV-seroconcordant partner (HIV-positive or HIV status unknown), and in only 0.3% (*n* = 608) was this the highest risk behavior (CLAI with ejaculation with a known HIV-positive partner).

There is a striking difference in the overall proportion of participants who reported CLAI with casual partners and the proportion of CLAI episodes that occurred with casual partners. At baseline, nearly 30% of participants in the Health in Men study reported any CLAI with casual partners (35). Due to the fact that most men (62.9%) who reported CLAI with casual partners engaged CLAI with casual partners occasionally (1–5 episodes) in the last 6 months (35), only <8% of the total CLAI episodes were reported with casual partners. A very limited number of studies have presented data on the number of CLAI episodes. This makes a direct comparison with similar samples of other HIV-negative men extremely difficult.

### Impact on HIV incidence

In the Health in Men study, each of the four risk reduction behaviors examined – serosorting, negotiated safety, strategic positioning, and withdrawal – was associated with an HIV incidence that was intermediate between that in those who reported no CLAI, and CLAI without that form of risk reduction behavior (34). In particular, negotiated safety and strategic positioning were not associated with significantly increased HIV incidence compared with no CLAI. However, serosorting with casual partners was associated with HIV infection rates about threefold higher than those who reported no CLAI, reflecting the less accurate knowledge about casual partner’s HIV status (36). Also, withdrawal was associated with a fivefold increased risk of HIV seroconversion, but this was confounded by the fact that it was commonly practiced with HIV-positive partners. Among those whose CLAI partners were HIV-positive, withdrawal was associated with a significantly lower risk than receptive CLAI with ejaculation (34).

It should be noted that the accuracy of the knowledge about a partner’s HIV status plays an important role in the effectiveness of serosorting and negotiated safety. In Sydney, the rate of HIV testing in gay and other men who have sex with men is among the highest in the world, and testing is most frequent in higher risk individuals (37, 38). These undoubtedly have contributed to the relatively stable HIV notifications in Sydney in recent years despite high overall levels of CLAI (39). Differences in HIV testing rates might explain why serosorting offers some degrees of protection in some settings (40), but not in others (41). In fact, mathematical modeling suggests that serosorting in casual sex settings will fuel HIV transmission in many settings where undiagnosed HIV is common (42).

### Recent advances in HIV prevention

Antiretroviral therapy reduces HIV transmissions by 96% in serodiscordant heterosexual couples, a strategy now known as treatment as prevention (TasP) (43). In serodiscordant male homosexual couples, interim results from the partner study showed no linked HIV transmission in 282 couple-years where the positive partner had undetectable viral load (44). Nevertheless, the interim partner study data could not exclude transmission occurring in as many as 1% of couples per year due to the relative short follow-up and thus limited statistical power. Full results from ongoing cohort studies in serodiscordant male homosexual couples, including the partner study in Europe and the Opposites Attract study in Australia, will be available around 2017 when the studies complete (45, 46). Despite the lack of conclusive evidence, some gay and other men who have sex with men appear to use undetectable viral load as one of the considerations when having CLAI with a HIV-positive partner, a phenomenon recognized as viral load sorting (23). Also, early evidence from the opposites attract study indicates that serodiscordant homosexual couples where the HIV-positive partner has undetectable viral load report more CLAI than couples where the viral load is detectable (47).

Recently, the iPrEX randomized controlled trial assessed the efficacy of PrEP use in HIV-negative men who have sex with men. This trial has been shown to be effective to reduce the risk of HIV acquisition by 96% when there are HIV drugs detected in the HIV-negative partner (7).

These new advances have heralded a new era of HIV prevention. Although there is no evidence of reduced condom use in studies among participants who believed they were taking antiretroviral drugs (48), it is inevitable that some gay and other men who have sex with men will embrace these new strategies as a means of circumventing the need for condom use while engaging in anal intercourse (49). The newly added options of TasP and PrEP will further complicate our understandings of whether CLAI or UAI is indeed “unprotected.”

The Health in Men study was completed in 2007, so the data presented may not represent current levels of sexual behaviors in the gay Australian community where the trends of serosorting and CLAI involving negotiation around HIV-positive partner’s viral load have since increased (50). However, this detailed analysis of a 7-year prospective study of all CLAI episodes demonstrates that vast majority of CLAI episodes are within the context of some form of HIV risk reduction. Participants in the Health in Men study were older (median age 35 years) and a high proportion reported a regular partner (57.8%) at baseline. In settings where HIV risk is mainly driven by younger gay and other men who have sex with men who are more likely to engaging in CLAI with casual partners (51), the pattern of risk reduction practice could be substantially different. Our definition of risk reduction behaviors was based on exclusive practice not on conscious intent. However, studies of intent are needed to elucidate the contexts of this decision making.

Using all forms of CLAI indiscriminately as a measure of HIV behavioral risk is no longer helpful in understanding the current drivers of HIV transmission in the community. Although CLAI remains the primary route of HIV transmission among gay and other men who have sex with men, it is essential to collect detailed information about relative risks associated with the various forms of CLAI, the impact of TasP and PrEP, and their trends overtime when conducting behavioral research. HIV behavioral campaigns aiming for an elimination of all CLAI risk are unlikely to be realistic in these changed circumstances and with greater knowledge about the range of non-condom-based risk reduction techniques. Many individuals in the community may increasingly view relying on condom use alone as unnecessary for HIV prevention. Continuous dialog between HIV researchers and gay communities involving accurate knowledge of current trends of CLAI and HIV risk associated with specific forms of CLAI would allow health campaign information to be developed in a tailored fashion to address the true drivers of HIV transmission.

## Conflict of Interest Statement

The authors declare that the research was conducted in the absence of any commercial or financial relationships that could be construed as a potential conflict of interest.
